# Tibial revision knee arthroplasty with metaphyseal sleeves: The effect of stems on implant fixation and bone flexibility

**DOI:** 10.1371/journal.pone.0177285

**Published:** 2017-05-08

**Authors:** Jan Nadorf, Stefan Kinkel, Simone Gantz, Eike Jakubowitz, J. Philippe Kretzer

**Affiliations:** 1 Laboratory of Biomechanics and Implant Research, Center of Orthopedics and Traumatology, University Hospital Heidelberg, Heidelberg, Germany; 2 Biometric Consulting and Project Management, Center of Orthopedics and Traumatology, University Hospital Heidelberg, Heidelberg, Germany; 3 Laboratory for Biomechanics and Biomaterials, Department of Orthopaedic Surgery, Hannover Medical School, Hannover, Germany; University of Memphis, UNITED STATES

## Abstract

**Introduction:**

Revision total knee arthoplasty often requires modular implants to treat bone defects of varying severity. In some cases, it may not be clear which module size and implant combination (e.g. sleeve and stem) should be chosen for a specific defect. When balancing implant stability and osseointegration against stress-shielding, it is important to choose an appropriate implant combination in order to match the given level of bone loss. Therefore, the necessity of stems in less extensive tibial defects and the advantage of different stems (lengths and stiffnesses) in combination with large metaphyseal sleeves on implant fixation and bone flexibility using a modular tibial revision knee system, were analyzed.

**Materials and methods:**

Four different stem combinations for a tibial revision implant (Sigma TC3, DePuy) were compared to an intact bone. Standardized implantation with *n* = 4 synthetic tibial bones was performed after generating an Anderson Orthopaedic Research Institute (AORI) Type T1 bone defect. Axial torques around the longitudinal stem axis and varus-valgus torques were separately applied to the implant. Micromotions of bone and implant were tracked using a digital image correlation system to calculate relative micromotions at the implant-bone-interface and bone deformation.

**Results:**

Overall, using stems reduced the proximal micromotions of tray and sleeve compared to no stem, while reducing bone deformation proximally at the same time, indicating some potential for proximal stress-shielding compared to no stem. The potential for increased proximal stress-shield due to reduced proximal deformation appeared to be greater when using the longer stems. The location of lowest relative micromotions was also more distal when using long stems as opposed to short stems. A short stem (especially a smaller diameter short stem which still achieves diaphyseal fixation) displayed less potential for stress-shielding, but greater bone deformation distal to the tip of the stem than in the natural model.

**Discussion:**

In the case of tibial revision implants with metaphyseal sleeves in a simple fully contained Type I defect, the absence of a stem provides for more natural bone deformation. However, adding a stem reduces overall relative micromotions, while introducing some risk of proximal stress-shielding due to increased diaphyseal fixation. Increasing stem length intensifies this effect. Short stems offered a balance between reduced micromotions and more proximal bone deformation that reduced the potential for stress-shielding when compared to long stems. A short stem with slightly smaller diameter (simulating a less stiff stem which still has diaphyseal fixation) increased the proximal bone deformation, but also tended to increase the bone deformation even further at the distal stem’s tip.

**Conclusion:**

In conclusion, further investigation should be conducted on fully contained Type I defects and the addition of a stem to offer better initial stability, taking into account stem length (i.e. shorter or more flexible stems) to support metaphyseal fixation and allowing bending found in intact bone. In addition, further study into more extensive tibial defects is required to determine if the stability/micromotion trends observed in this study with stems and sleeves in Type I defects still apply in cases of extensive proximal bone loss.

## Introduction

### Background of revision total knee arthroplasty (TKA)

Total joint arthroplasty, especially for hip and knee joints, is one of the most successful therapies in modern medicine. The aims of this therapy include the reduction of pain, regaining functionality as well as mobility and a faster integration into the patient’s daily life. However, implant failure can occur [1–3], thus requiring a revision surgery in which the whole implant or single parts must be replaced. Regarding knee joints, long-term outcome of revision implants (e.g. 87.4% after 5 years [4]) is less favorable when compared to primary surgeries (e.g. 94.9% after 10 years [2]). This could be caused by a more complex clinical situation during revision surgery, as in most revision cases extended bone defects exist. Given the great variety of bone defects, customizable implants are required to treat patients in the best possible way.

### Clinical application of modular implants in revision TKA

Modular implant systems make it possible to address most clinical situations.—For example, in the case of proximal tibial reconstruction with small bone defects, a tibial tray could be used. Larger defects or a decreased quality of bone tissue might require the adaption of proximal augments, metaphyseal sleeves or diaphyseal stems to bridge the deficient bone and enable a better fixation of the implant in a more healthy bone area. Although defect classifications with treatment suggestions exist [5–8], the orthopedic surgeon must choose the optimal modular combination depending on each specific clinical situation. Metaphyseal sleeves offer the surgeon the option of including proximal biological fixation as part of their revision tibial fixation strategy.–Having a greater biomechanical understanding of the relative importance of metaphyseal and diaphyseal fixation for overall fixation in different types of defects would help guide clinicians’ fixation choices.

### Basics of implant fixation and implant loosening

A decisive factor for the long-term outcome of joint replacement is the implant stability, which is based on the initial press-fit of the implant in the bone achieved during surgery [9]. However, the local bony contact areas are subjected to natural bone remodeling [10], and therefore may weaken with time [9]. Wolff’s law states, that depending on loading, bone tissue will remodel itself in favor of better load transmission [11]. Bone tissue that is intensively loaded will be strengthened by osteoblasts, and bone with less intensive loading will be reduced by osteoclasts [10, 12, 13]. Insertion of a knee implant massively changes the load transmission within the tibial bone. To achieve biological fixation, the load transfer from implant to bone should have osseointegration of bone cells into the synthetic knee’s porous fixation surfaces; however, an imbalance of osteoblasts and osteoclasts could result in implant loosening [9]. In spite of these biological and biomechanical processes, mechanical stimuli could also influence bone ingrowth. It has been shown that extensive relative motions of 150 μm might destroy already built bony bridges and form a fibrous connective tissue at the implant-bone surface, which complicates osseointegration in the afflicted area [14–16]. Aseptic loosening as a result of any of these processes can occur, which has been found to be a major cause of implant failure [17].

### Current state of clinical and experimental research

Based on the variety of modular options and individuality of the patients and bone defect, only a few relevant clinical studies are available regarding biomechanical behavior of modular knee prostheses. For example, the number of observed patients per study can vary from 60 [18] to 1512 [19]. Subjective evaluation of X-rays [18–20] and pain scores [18, 19, 21, 22] are dependent on observer and patient; therefore, they are not easily comparable. Likewise, a statistical comparison with alternating defect situations at the moment of surgery is complicated. Furthermore, information regarding daily activities of the patient and consequently, implant loading is unknown in these studies. Therefore, these studies are difficult to transfer to multiple implant systems or modular combinations.

However, experimental studies can be highly standardized and can be compared to a large number of implant groups. Since osseointegration is generated over time in patients, it cannot be analyzed in vitro. Therefore, in-vitro studies have mainly focused on analyzing the primary stability of an implant, as an important basis for good, long-time fixation [9]. As stress and micromotion seem to be a decisive factor for implant failure, the focus for implant fixation analyses is on these parameters. Both variables can be measured precisely with experiments. For example, some biomechanical studies measured implant related stress with strain gauges [23–26] or implant-bone relative micromotions with inductive sensors [23, 26, 27]. However, implant fixation and bone flexibility of tibial revision knee implant modularity with regards to using sleeve and stem fixation individually and in combination has not yet been analyzed.

As already stated, surgeons in revision TKA are confronted with complex bone defects and have the option of using different implant modules, which can lead to different clinical interactions. One option to treat a metaphyseal defect that has sufficient cortical bone tissue is to use large proximal sleeves without additional distal stabilizing modules. An additional distal stem may increase implant stability; however, this could be accompanied by the simultaneously increased risk of proximal stress-shielding. Also, the surgeon must take the natural bone flexibility into account, as a stem could stiffen the implant-bone compound. Shorter or undersized stems (one size thinner in diameter but still canal-filling) offer an additional compromise, which is somewhere between proximal load transmitting and distal stabilizing.

### Aims

Therefore, the aim of this study was to analyze the implant fixation and bone flexibility of four modular combinations of a tibial revision knee system. The following hypotheses were made:

1. If tibial stems are added while using large metaphyseal sleeves in revision TKA, proximal implant fixation will decrease and load behavior will most closely resemble the behavior of an intact bone.
2. If short stems are used instead of long stems, proximal implant fixation will increase and load behavior will most closely resemble the behavior of an intact bone.
3. If stems with undersized diameters are used instead of press-fit stems, load behavior will most closely resemble the behavior of an intact bone.

## Materials and methods

### Bones and implants

**Fig 1:**
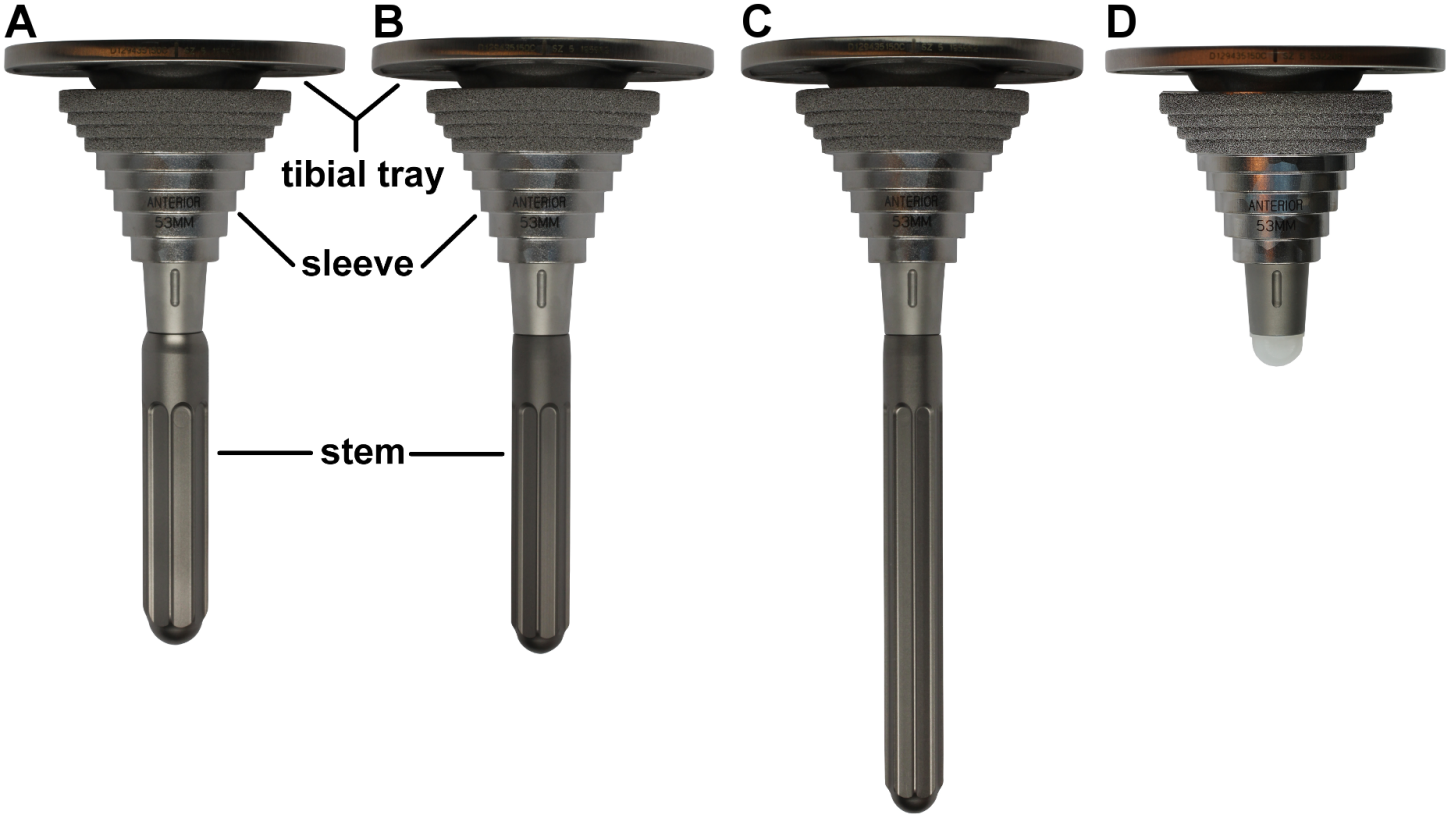
Implant groups (A: short press-fit stem, B: short undersized stem, C: long press-fit stem, D: no stem), each combined with comparable M.B.T. Revision tray and sleeve.

Twenty synthetic tibial bones (composite bone 4^th^ generation (#3402), Sawbones Europe, Malmö, Sweden) were divided into five groups with *n* = 4 each. The clinically used tibial revision system Sigma TC3 (DePuy Synthes, Warsaw, USA), was chosen for the four implanted groups (Group A-D, Fig 1). Another group (Group E) was used to track the motion of an intact bone and served as a control of physiological conditions.

**Fig 2:**
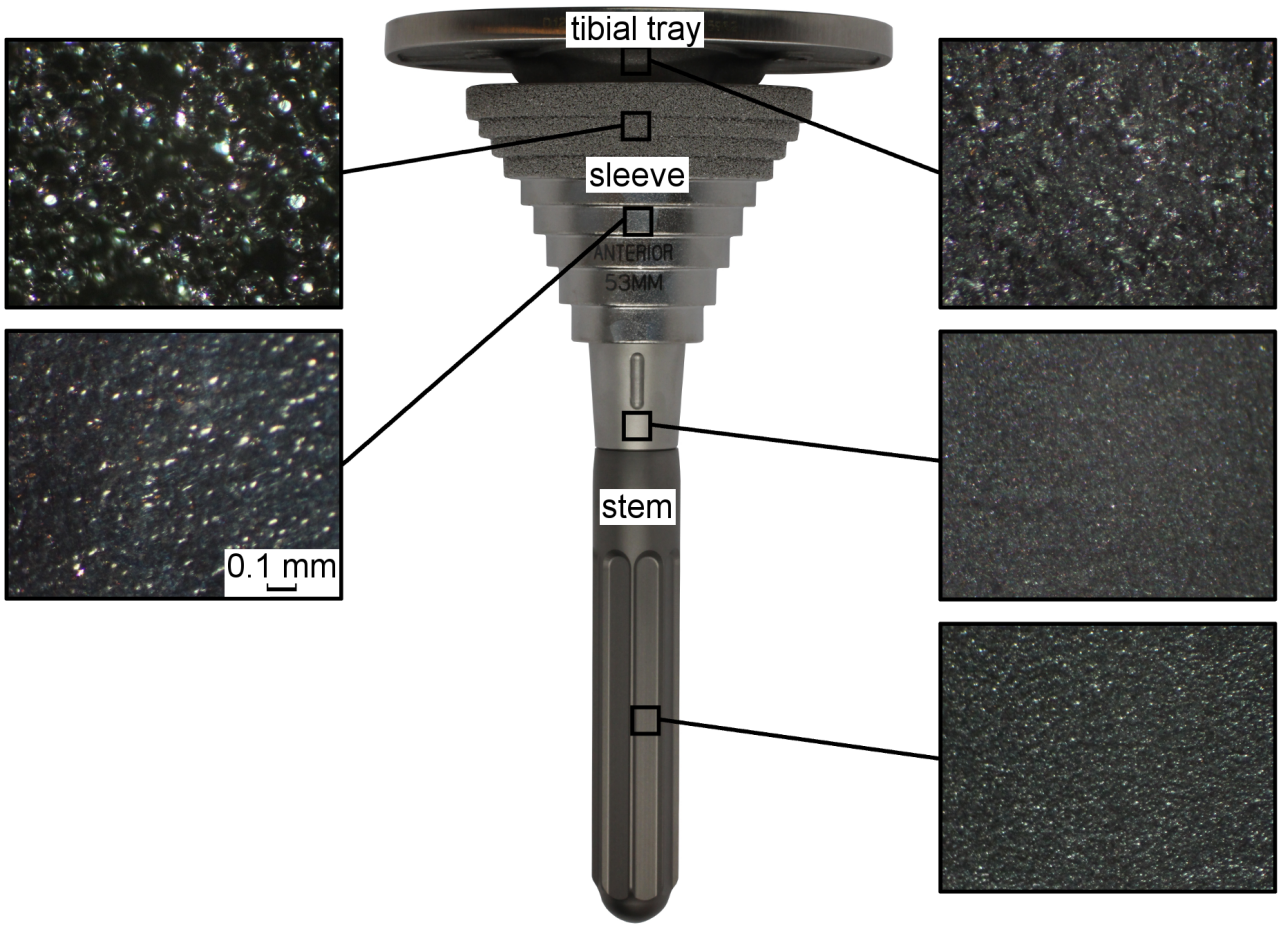
Microscopic and macroscopic surfaces of a tibial tray, combined with sleeve and distal stem.

Each group contained a cemented mobile bearing tibial tray (M.B.T. Revision, rotating platform, size 5), which was manufactured from a cobalt chrome alloy (CoCrMo) following ASTM standard F-75. The proximal part of the conical hollow intramedullary stem and the distal surface of the tibial plateau are grit blasted to enhance the cement fixation (Fig 2). Additional fixation stability-enhancing or bone defect treating sleeve components (e.g. augments, sleeves and stems) can be attached. Within this study, a large cementless proximal sleeve (M.B.T. Revision, size 53 mm) made of a titanium alloy (TiAl6V4) was used for each implant construct (Fig 2). The outer surface of the oval-shaped terraces is proximally porous coated to provide additional fixation stability and induce proximal biological ingrowth. The polished surface should prevent faster distal ingrowth vs. proximal bone ingrowth from happening. Four different cementless stem options were compared: Group A: short press-fit stem (P.F.C. Sigma modular fluted rod, 75 mm size 16); Group B: short undersized stem (P.F.C. Sigma modular fluted rod, 75 mm size 14); Group C: long press-fit stem (P.F.C. Sigma modular fluted rod, 115 mm size 14); Group D: no stem. All cylindrical stems were manufactured from a titanium alloy (TiAl6V4) without a specific surface modification; however, they were longitudinally fluted, except for the proximal 20 mm (Fig 2). The distal end of the stems is spherically narrowed.

### Preparation

**Fig 3:**
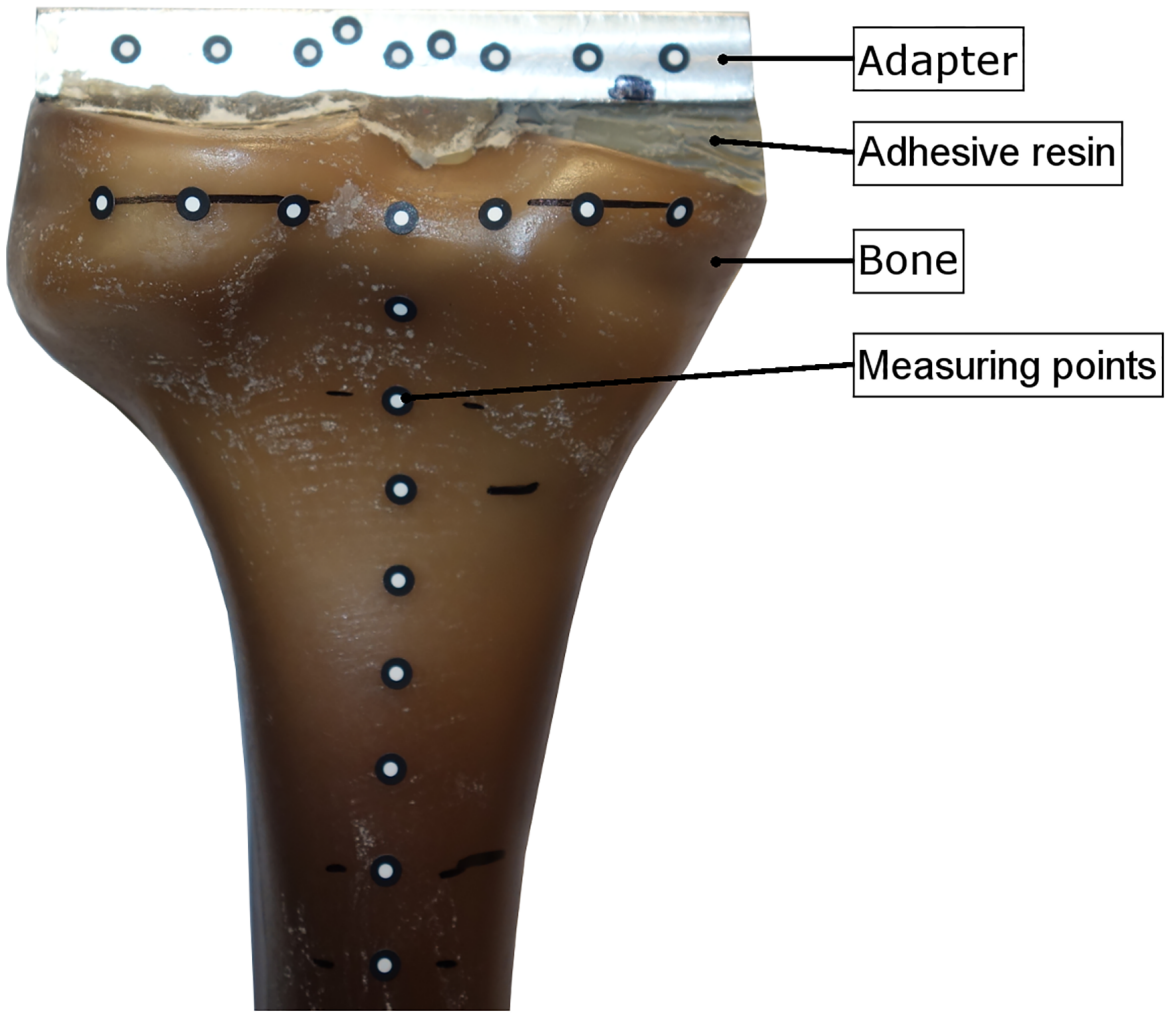
Connection of an adapter to the intact bone using a template and adhesive resin.

**Fig 4:**
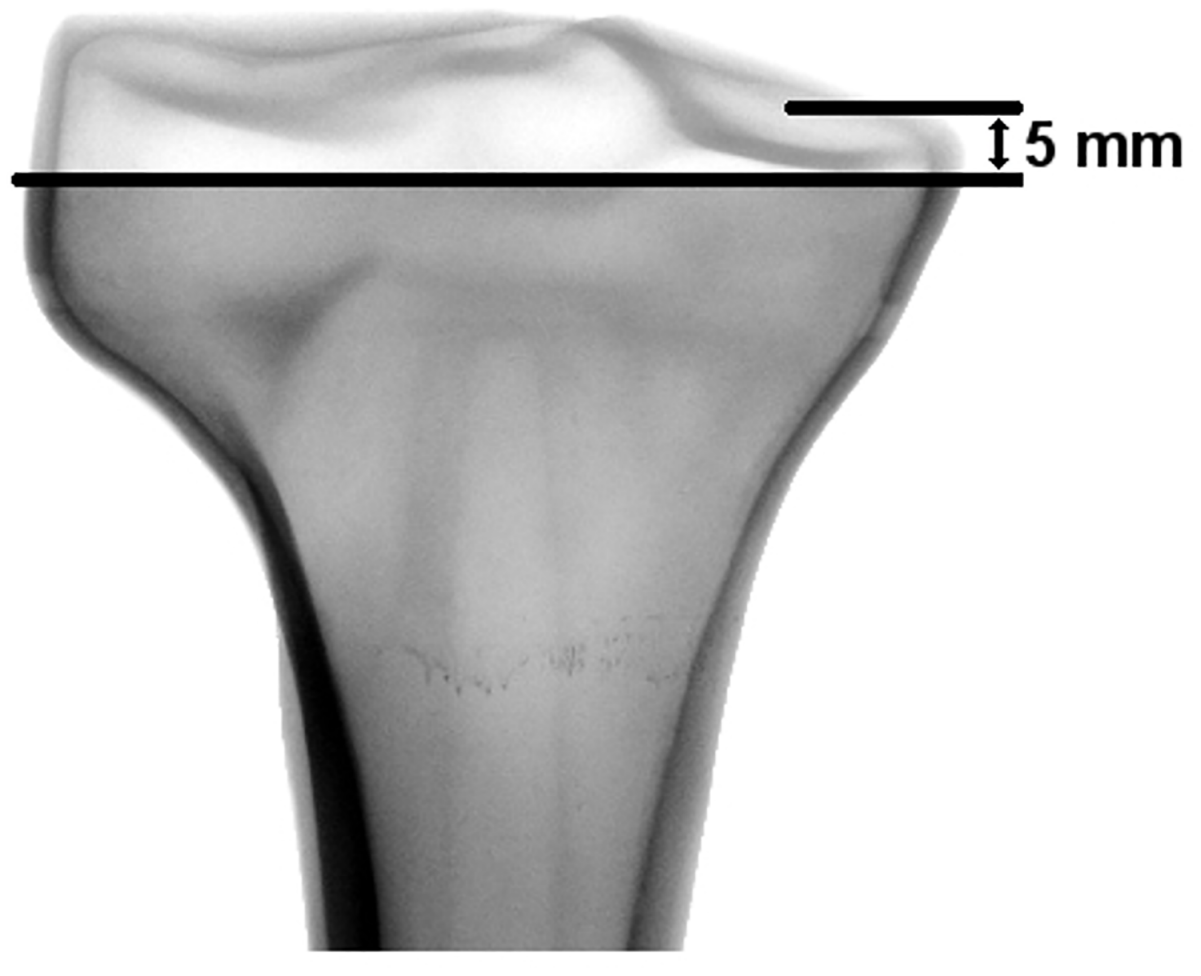
Simulated AORI Type I resection using a template, located 5 mm distal to the medial plateau.

An adapter was attached with an adhesive resin to the proximal tibial plateau of the intact bones without any resection or preparation of the bones (Fig 3, Group E). Within all other groups, a standardized 5 mm resection of the proximal tibial plateau was performed, measured from the medial plateau using a template. Implant sizes were planned by means of synthetic bone radiographs. The implanted groups were prepared by an experienced surgeon in a standardized manner using the implant-specific instruments. The cortical shell was left intact to simulate AORI Type I tibial bone defects (Fig 4) [28]. All bones were rigidly fixed in the distal part at the same level by polyurethane (Rencast^®^ FC 53, Huntsman GmbH, Bad Saekingen, Germany) with a distance of 137.5 mm to the distal end of the implant in case of the long stem. Regarding cement penetration, the spongeous bone structure resembled sclerotic bone areas. Following clinical application [29], twelve small holes (diameter 3 mm, depth 5 mm) were drilled into the proximal tibia plateau using a custom made template.

To reconcile the gap between the irregular proximal bone surface and implant, bone cement (SmartSet MV, DePuy Synthes, Warsaw, USA) was applied according to the manufacturer’s instructions. Using a material testing machine (Frank-Universalpruefmaschine 81816/B, Karl Frank GmbH, Weinheim-Birkenau, Germany), the sleeves were mounted to the tibial tray with F = 5000 N and the implants were pressed into the synthetic bone with a load of F = 2000 N within the period of bone cement polymerization in order to resemble the surgeons’ pressure on the implant and avoid malposition of the implant. The implant fit was controlled by X-ray examination.

### Description of the test setup

**Fig 5:**
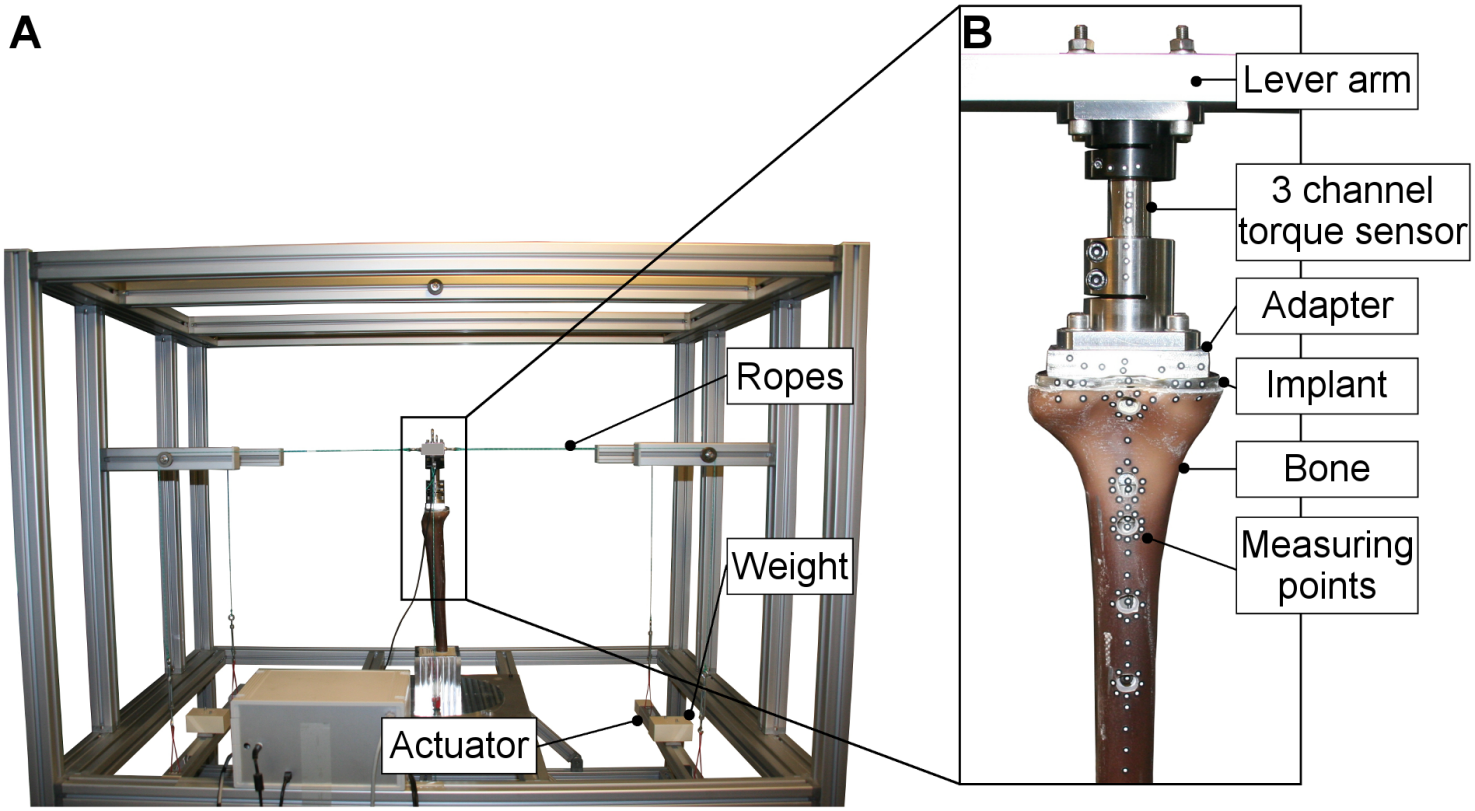
(A) High-resolution measuring device: entire setup used and (B) details of the measuring area.

Implant fixation and bone flexibility were determined using a custom-made high-resolution measuring device that had been established in previous studies [30–34] (Fig 5). Thus, different loads could be applied on the implant by using a rope system with shifting weights.

**Fig 6:**
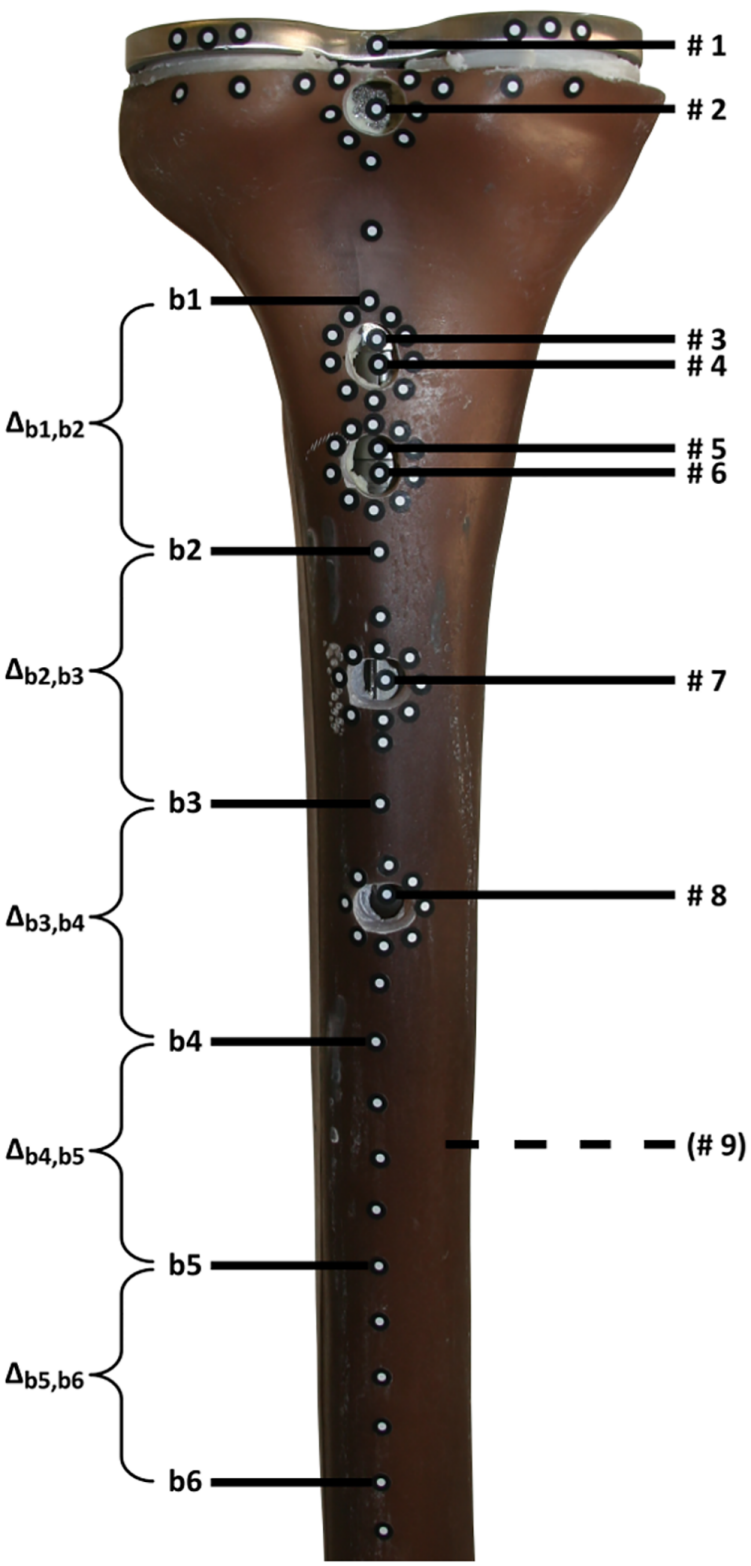
Location of the implant stability measuring points #1-#9 and bone flexibility measuring points b1-b6 as well as deformation sections Δ_b1,b2_ to Δ_b5,b6_.

**Fig 7:**
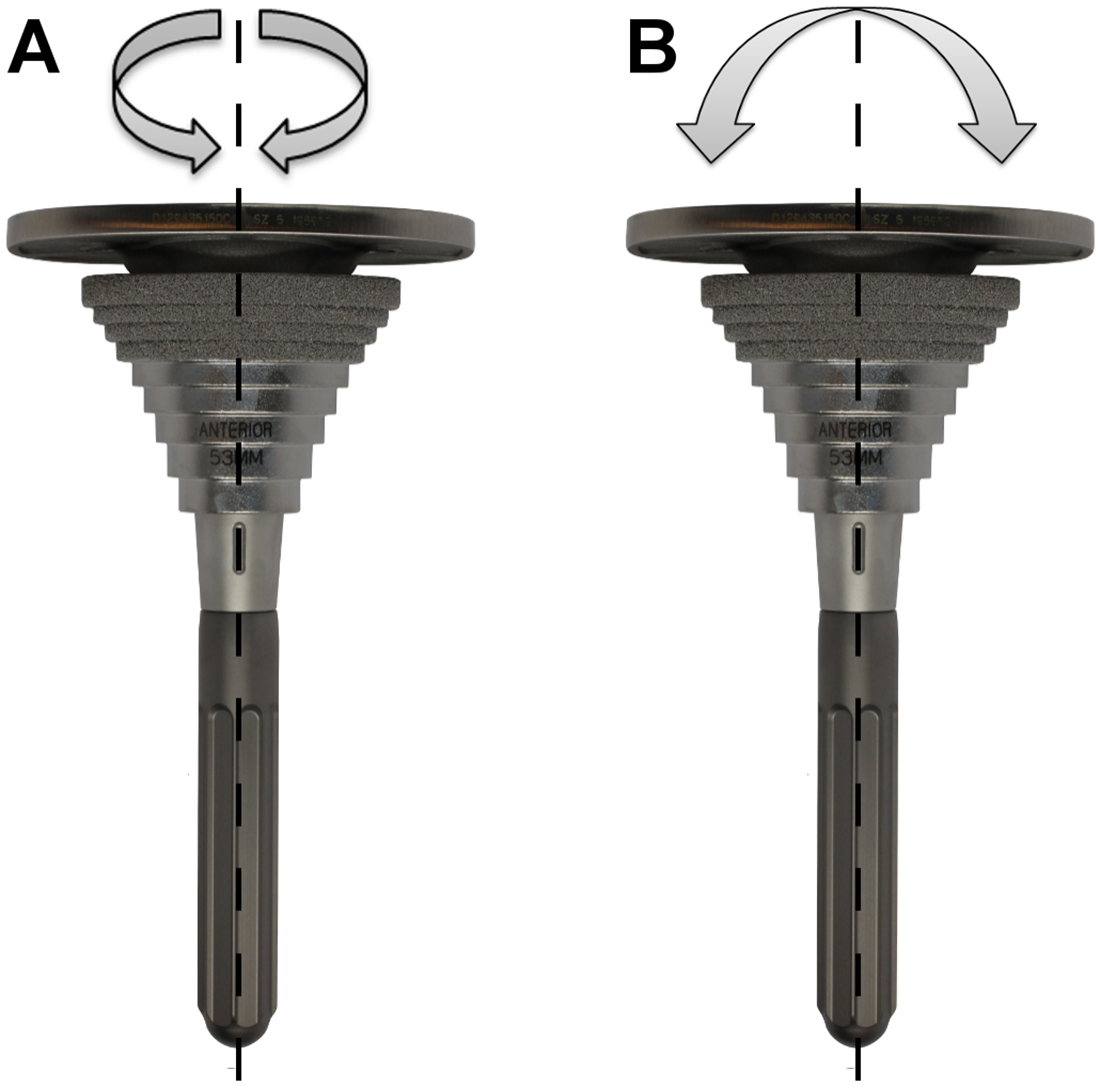
(A) Schematic of the applied axial torque around the longitudinal stem axis and (B) of the medio-lateral torque.

The spatial micromotions of the synthetic bones and the implants were investigated at multiple locations (Fig 6) under two separate cyclic load applications using a digital image correlation (DIC) system (Pontos, GOM, Braunschweig, Germany) with an accuracy of 1 μm. As a linear correlation between implant loading and implant-bone relative micromotions has been shown during axial torque application in an experimental setup [30–33], reduced loads compared to standardized loads occurring in knee implants [35] were used to resemble the initial regeneration phase and to allow reproducible non-destructive measurements. At first, an axial torque along the longitudinal stem axis of TZ = ±7.0 Nm was applied, followed by a medio-lateral torque of TX = ±3.5 Nm, which has been established for primary implant stability analysis (Fig 7) [27, 30, 36].

The optical measurement device has an incremental trigger to take images every 0.25 Nm independent of load application speed. Each measurement contained six load cycles. To track the spatial motion of the implant, nine points of interest along the longitudinal axis were measured from dorsal side (Fig 6). Three points were located on the tibial tray (# 1: proximal tibia plateau, # 4: near to distal sleeve, # 5: near to proximal stem), two on the sleeve (# 2: proximal, # 3: distal), and the other four on the stem (# 6: proximal, # 7: middle, # 8: distal end of short stem, # 9: distal end of long stem). Due to the absence of a stem, the no stem Group D did not need the measuring points # 6 - # 9, just as the short stem Groups A and B did not need the measuring point # 9. Small holes were drilled into the bone to access the implant surface. Consequently, the bone motion could not be measured at these points; however, measuring the motion of multiple points at the rim of each drilled hole provided an approximate best-fit point, which was also projected on the implant surface.

Every measuring point is related to the global coordinate system of the camera. The relation between absolute micromotions of the implant measuring points and the corresponding tibia measuring points results in implant-bone relative micromotions. These relative micromotions at the implant-bone-interface allow for the identification of the rotational and bending implant stability and for the determination of the fixation modus of the stem. As previously stated, extensive relative motions of 150 μm at the implant-bone-interface may complicate osseointegration [14–16]. Bergmann et al. showed maximal torsional loads of 18.9 Nm during walking and maximal varus-valgus loads of 59.1 Nm in case of descending stairs occurring in knee implants [35]. Converted to the reduced loads (7 Nm torsional load and 3.5 Nm varus-valgus load) in this study, a threshold of (7 Nm * 150 μm) / 18.9 Nm = 56 μm during rotation load application and of (3.5 Nm * 150 μm) / 59.1 Nm = 9 μm during varus-valgus load application would be of interest for osseointegration.

Unlike the loads which were reduced necessarily in the case of implant fixation measurements, a more physiological [35] medio-lateral torque of TX = ±20 Nm was applied for bone flexibility analysis. In this case, the torque is appropriate because it simulates when the patients begin to put the joint under full loading. To compare the deformation of implanted groups to the deformation of an intact bone, six additional measuring points b1-b6 were used (Fig 6). They were located 40 mm (b1), 80 mm (b2), 120 mm (b3), 160 mm (b4), 200 mm (b5) and 240 mm (b6) distal to the adapter. Relative micromotions (Δ_b1,b2_, Δ_b2,b3_, Δ_b3,b4_, Δ_b4,b5_ and Δ_b5,b6_) of all groups were measured. The bending flexibility of the bone can be identified by comparing these motions to those of the intact bone deformation of Group E.

### Statistics

Data from a similar experimental setup [36] were used for an a priori sample size estimation (decisive power of 0.80) with a statistical power analysis tool (G*Power version 3.1.9.2, University Duesseldorf, Germany). When using analysis of variance (ANOVA) to determine the biomechanical implant fixation of a single stem or to compare the biomechanical implant fixation of different stem designs, a group size of *n* = 4 was found to be sufficient. However, regarding this sample size, additional post-hoc power analyses with ANOVA were performed to verify the power of the results. Independent of implant group and load conditions, a power of 100% was achieved for characterization of implant stability and a power of 96–100% was achieved for analysis of bone deformations.

ANOVA was used to compare the relative micromotions within each implant group for characterization of implant stability. Motions at the same measuring level of two different implant groups were analyzed with Student’s T-test for unpaired samples. The influence of an implant on bone deformation can be shown when comparing the relative micromotions of different groups also using ANOVA. Bone deformation at the same area from two different groups was compared with a Student’s T-test. A value of p ≤ 0.05 was considered to be significant. All measured data were shown as mean and standard deviation (±SD). All calculations were performed using SPSS (Version 22.0. Armonk, NY: IBM Corp).

## Results

### Rotational and bending implant stability

**Fig 8:**
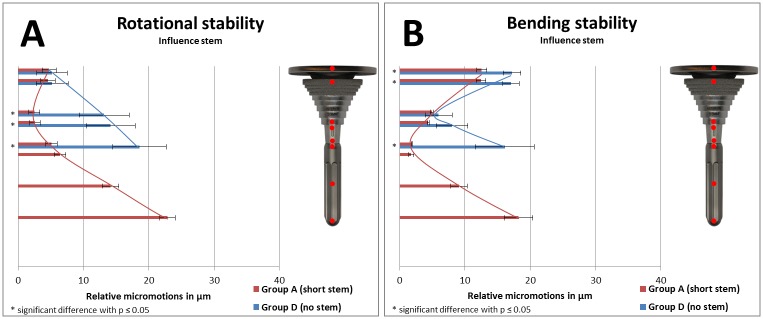
(A) Rotational implant stability and (B) bending implant stability based on measured relative micromotions between bone and implant. Comparison of Group D (no stem, depicted in blue, measuring points #1-#5) vs. Group A (short press-fit stem, depicted in red, measuring points #1-#8).

**Fig 9:**
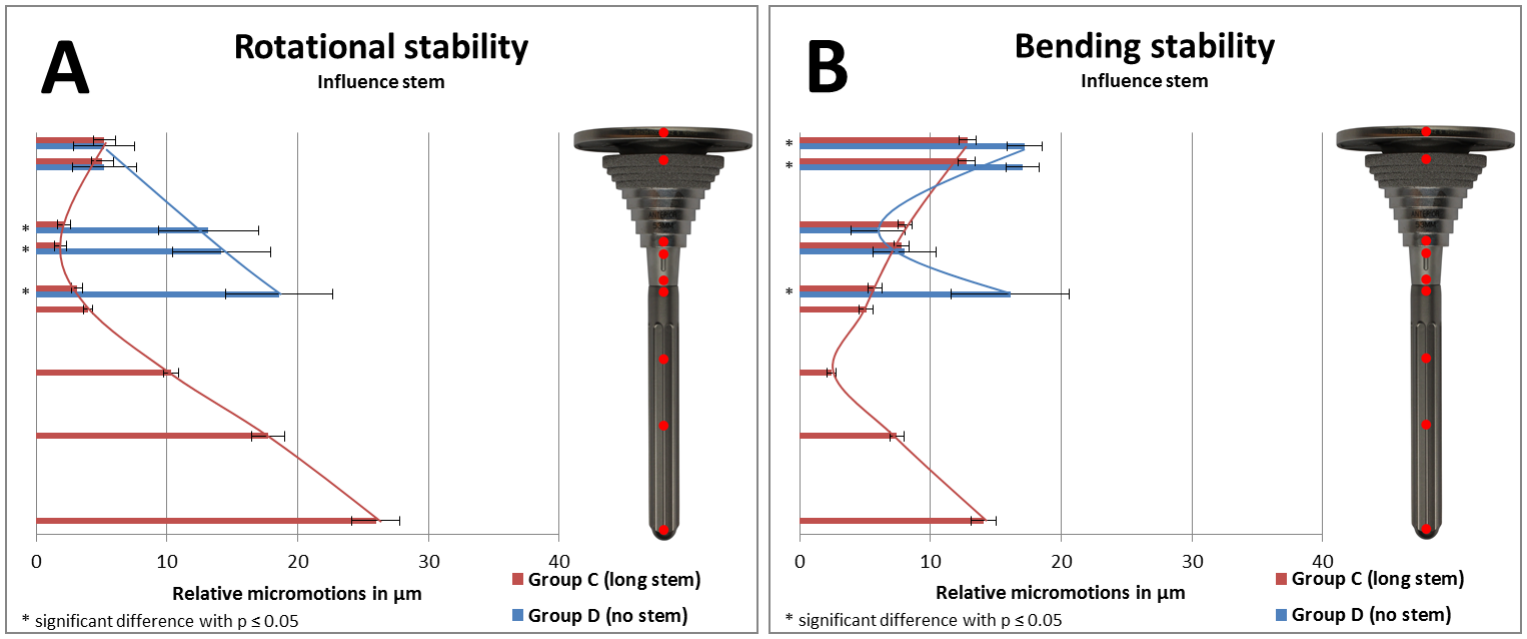
(A) Rotational implant stability and (B) bending implant stability based on measured relative micromotions between bone and implant. Comparison of Group D (no stem, depicted in blue, measuring points #1-#5) vs. Group C (long press-fit stem, depicted in red, measuring points #1-#9).

**Fig 10:**
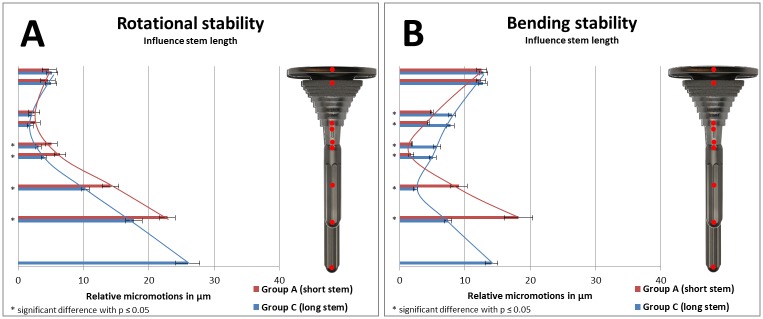
(A) Rotational implant stability and (B) bending implant stability based on measured relative micromotions between bone and implant. Comparison of Group A (short press-fit stem, depicted in red, measuring points #1-#8) vs. Group C (long press-fit stem, depicted in blue, measuring points #1-#9.

**Fig 11:**
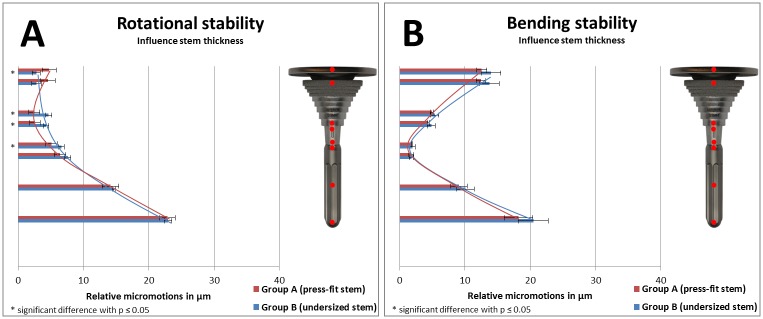
(A) Rotational implant stability and (B) bending implant stability based on measured relative micromotions between bone and implant. Comparison of Group A (short press-fit stem, depicted in red, measuring points #1-#8) vs. Group B (short undersized stem, depicted in blue, measuring points #1-#8).

Relative micromotions between implant and bone as a result of implant stability of specific groups were compared in Figs 8 and 9 (to analyze the influence of a stem), in Fig 10 (to analyze the influence of stem length), and in Fig 11 (to analyze the influence of stem diameter) with bar charts. Relative micromotions were plotted in μm from proximal to distal for all measuring points #1 to #9 of the implanted Groups A-D.

All measured relative micromotions were below the threshold regarding rotational implant stability. In addition, the motions in the area of main fixation during varus-valgus torque were also below the threshold for osseointegration in all groups. However, proximal and distal to the area of main fixation, the threshold of 9 μm was exceeded in all groups during varus-valgus load application.

During axial torque application, highest relative micromotions were measured at the distal part of all implants. However, the location of lowest relative micromotions differed between the groups from the distal end of the sleeve (Group A and Group C) to the proximal end (Group B and Group D). During varus-valgus torque application, highest relative micromotions were measured at the distal part for both short stem groups, at the proximal part when using no stem and with equally proximal/distal distribution in the long stem group. Lowest relative micromotions differed between the groups from proximal end of the stem (Group A and Group B) to the middle area of the stem (Group C) and to the distal part of the sleeve (Group D). Independent of loading condition, standard deviation was increased in the group without distal stem.

Comparing rotational stability of Group D with either short stem Group A (Fig 8) or long stem Group C (Fig 9) resulted in increased relative micromotions for the no stem group in the distal part of the sleeve and the tibial tray (each comparison *p≤0*.*01*). Comparing bending stability of Group D with either short stem Group A (Fig 8) or long stem Group C (Fig 9) resulted in increased relative micromotions for the no stem group in the proximal part of the tibial tray and the sleeve as well as for the distal part of the tibial tray (each comparison *p<0*.*01*).

Using long stems vs. short stems reduced relative micromotions along the full length of the stem regarding rotational implant stability testing (Fig 10, each comparison *p≤0*.*03*). During bending stability testing, distal relative micromotions were reduced as well when using longer stems (Fig 10, each comparison *p<0*.*01*). However, compared to the short stem group, increased relative micromotions were measured for the longer stem in the distal part of the sleeve and tibial tray (Fig 10, each comparison *p<0*.*01*).

When undersized stems were used instead of press-fit stems (Fig 11), increased relative micromotions were measured during rotational implant stability testing in the distal part of the sleeve and the tibial tray (each comparison *p≤0*.*04*). However, regarding bending stability, no relevant differences was measured between both short stem groups.

### Bending bone deformation

Relative micromotions of the bone as a result of bone deformation (Table 1) were given in μm for the measured bone sections (Δ_b1,b2_, Δ_b2,b3_, Δ_b3,b4_, Δ_b4,b5_ and Δ_b5,b6_) of all groups. Highest relative micromotions were measured in the distal sections Δ_b3,b4_ to Δ_b5,b6_ when using implants with stems (independent of stem length or side distribution of the applied load). An implant without stem results in a more uniform distribution of relative micromotions with the lowest deformation in section Δ_b4,b5_. The measured micromotions of the intact bone are more uniform as well when compared to groups A-C, with highest motions in section Δ_b3,b4_ and lowest motions in the proximal area.

**Table 1:** Measured relative micromotions at the bone sections Δ_b1,b2_ to Δ_b5,b6_ are shown for intact bone Group E and for all implant groups (A-D) described as means and standard deviation (±SD). The applied varus-valgus torque is separated into lateral and medial load condition to analyze bending bone deformation.

Group		Varus-valgus torque: Relative micromotions in μm									
		Lateral load					Medial load				
		Δ_b1,b2_	Δ_b2,b3_	Δ_b3,b4_	Δ_b4,b5_	Δ_b5,b6_	Δ_b1,b2_	Δ_b2,b3_	Δ_b3,b4_	Δ_b4,b5_	Δ_b5,b6_
A	*mean*	-3	-5	-11	-8	-10	1	5	10	9*	11*
	*±SD*	*2*	*2*	*3*	*3*	*2*	*2*	*2*	*4*	*2*	*3*
B	*mean*	-4	-7	-13*	-10	-11	3	7	12*	9*	12*
	*±SD*	*2*	*2*	*3*	*3*	*3*	*1*	*1*	*4*	*2*	*3*
C	*mean*	-2	-3*	-4*	-6	-7	1*	2*	5	3	5
	*±SD*	*2*	*2*	*2*	*2*	*1*	*1*	*1*	*1*	*2*	*1*
D	*mean*	-6	-6	-6*	-4*	-5	4	4	5	3	5
	*±SD*	*1*	*1*	*1*	*2*	*1*	*1*	*1*	*2*	*1*	*2*
E	*mean*	-4	-7	-8	-7	-7	3	5	6	4	4
	*±SD*	*1*	*2*	*1*	*2*	*2*	*1*	*2*	*1*	*1*	*1*

Significant differing deformation in a specific bone section vs. Group E with *p<0*.*05* are highlighted with a “*”.

**Fig 12:**
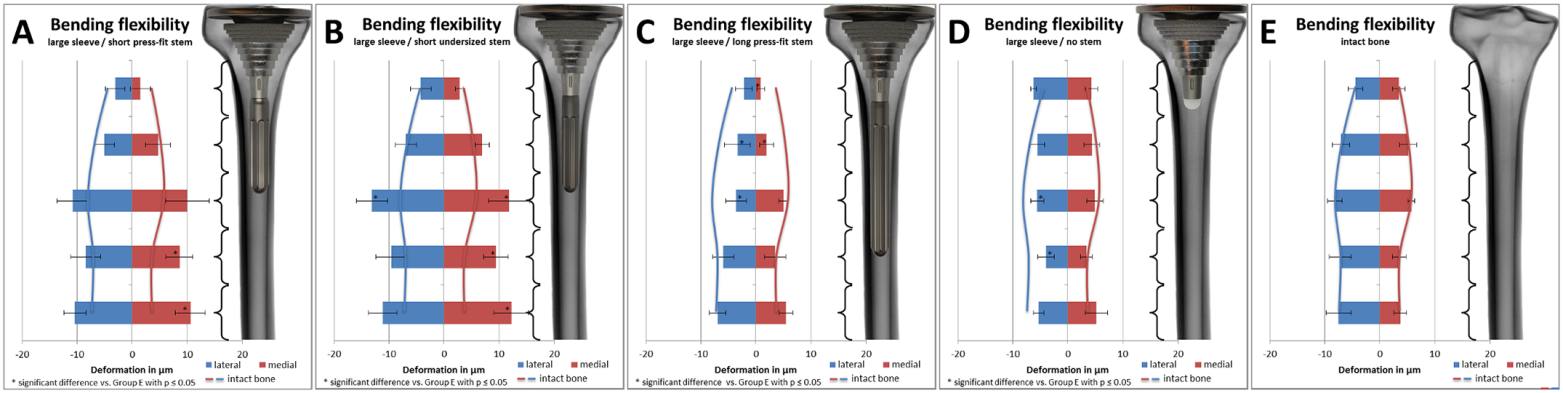
Bone flexibility of Groups (A-E) in five different sections of the tibia during varus-valgus load application (separated into lateral ~blue and medial ~ red). Deformations are given as relative micromotions between six bone measuring points in μm. Bar charts show the deformation of each group, while continuous lines resemble the deformation of an intact bone. Higher motions are accompanied by higher flexibility of the bone-implant compound and lower motions are accompanied by lower flexibility of the compound.

Bone flexibility results are given in μm with standard deviation and are summarized in Fig 12. Differences between the measured absolute micromotions were calculated and are shown in bar charts for all groups. In addition, to compare the bone deformation of the implant Groups (A-D) to the deformation of an intact bone, the results from Group E are shown by lines in every bar chart. Increased motions were accompanied with higher flexibility of the bone and lesser motions were accompanied by stiffening of the bone by the implant.

During varus-valgus torque application, bone deformation of the intact bone (Group E) differed from the implanted groups. Motions were distally decreased during lateral load application for the no stem group (Group D vs. Group E, *p≤0*.*04*), whereas increased distal motions were measured when using a short stem (Group A vs. Group E during medial load, *p≤0*.*02* and Group B vs. Group E during lateral and medial load, *p≤0*.*02*). When using a long stem, proximal relative micromotions were decreased independent of load application (Group C vs. Group E, *p<0*.*05*). Furthermore, no difference in micromotions was measured with varying stem diameters. When using a stem, increasing the stem length decreased distal relative micromotions during lateral and medial load application (Group A vs. Group C, *p<0*.*05*). Compared to an implant without stem, adding a short stem proximally reduced the motions during lateral load application (Group A vs. Group D, *p≤0*.*03*) and distally increased the measured micromotions during lateral and medial load application (Group A vs. Group D, *p≤0*.*04*), while adding a long stem only increased proximal motions during both load applications (Group C vs. Group D, *p≤0*.*04*).

## Discussion

### Background and rationale

Revision TKA is a complex procedure that yields less favorable outcomes than primary surgeries [2, 4]. Modular implant systems are beneficial in treating diverse bone defect situations; however, an increased modularity requires that the surgeon consider the disadvantages and advantages for each possible implant combination. When deficient bone tissue is present at multiple locations, choosing an appropriate implant that is as small as possible yet as large as necessary can be quite difficult. As aseptic loosening is a major reason for implant failure in revision TKA [17], its’ causes and effects (e.g. bone remodeling [10, 12, 13], osseointegration [11, 37, 38], stress-shielding [1, 25, 39], etc.) should also be well known and considered in the selection of appropriate module combination.

In case of a moderate metaphyseal defect with deficient cancellous bone and sufficient cortical bone, an implant with a metaphyseal sleeve and diaphyseal stem would be recommended. However, a distal supporting stem can be accompanied by a more distal implant fixation, thus increasing the risk of proximal stress-shielding. How the biomechanical behavior of a tibial modular implant with metaphyseal sleeve could be improved in such a defect situation was a point of interest. Therefore, it was analyzed whether the absence of a diaphyseal stem or reduction in stem length or stiffness might be beneficial for the fixation and load behavior.

### Discussion: (1) If tibial stems are added while using large metaphyseal sleeves in revision TKA, proximal implant fixation will decrease and load behavior will most closely resemble the behavior of an intact bone

In the current study, using a tibial implant with a metaphyseal sleeve and diaphyseal stem vs. using only a sleeve reduces relative micromotions for rotational and varus-valgus loads, which could be beneficial for osseointegration. Areas of lowest micromotions are extended to implants with stems, which could be beneficial for load transmission from implant to bone and may reduce the risk of load peak induced fractures. Especially during varus-valgus load application, adding a stem helps to not exceed critical values for osseointegration in the greater section of the implant. Nevertheless, main fixation area of implants with stems, vs. implants without stems, is found to be more distal. Therefore, the risk of proximal stress shielding induced atrophy could be increased. However, the missing stem is accompanied by missing intramedullary guidance during the reaming process, which increases the risk of tilting and may be responsible for the increased overall standard deviation in the group without stems. (CAVEAT: The use of the tested tibial revision implant with sleeve but without a stem has not yet been developed by the manufacturer; therefore no surgical instruction existed for this case.) Thus, a mechanical intra- or extramedullary guide is recommended to achieve the correct alignment in this case.

Just as with implant stability, the presence of a stem influenced implant-bone flexibility during varus-valgus load application. The bone flexibility decreased distally when using implants without stems, compared to an intact bone. Proximal flexibility of an implant without stem may be comparable to the intact bone, as the resection of the proximal tibial plateau seems to disturb the stability of the tibia and thus increases the bone flexibility before it is going to be stiffened by the implant. In contrast, bone flexibility decreased proximal and increased directly distal to the stem when using implants with stems. Nevertheless, none of the used implants restored the flexibility of an intact bone.

In a prospective study with 121 patients and 193 implanted tibial and femoral sleeves, Graichen et al. [40] analyzed clinical and radiographic data. A 98.3% aseptic overall survival rate was observed for a mean follow-up of 3.6 years. Off-label used stemless implants were included in this study although they were only used in few cases to avoid malalignment; however, it was considered that the sample size was too small to yield any conclusive results about the stemless fixation. Other biomechanical studies have proved the advantages of stems in revision TKA and have shown an increased mechanical stability due to a larger load transferring area accompanied by reduced stresses at the bone [41, 42]. In the current study, similar results have been shown, which is demonstrated by the proximally increased area of low relative micromotions in the stemmed groups, compared to the group without diaphyseal stem. In an experimental study with thirty human tibial specimens, Stern et al. [43] analyzed the axial migration of tibial knee implants with different stem lengths and cemented as well as cementless fixation during three different axial pushing load scenarios. After excluding twenty test objects because of testing complications, no results indicating an enhanced implant fixation were measured, independent of stem length or cementing option. However, Stern et al. [43] observed a clear trend that cemented stems have a stabilizing effect compared to cementless stems and that implants with longer stem have a poorer proximal fixation compared to stemless implants. Instead of the mentioned axial migration, Nadorf et al. [34] analyzed the initial fixation of a tibial knee implant during varying load scenarios. In the presence of an extended AORI Type 2a bone defect, adding a diaphyseal stem to a tibial implant with proximal sleeve reduced metaphyseal micromotions and thus supported a fixation around the sleeve. Compared to an intact bone, an implant with stem vs. an implant without stem provides a more natural bone deformation.

Based on the biomechanical studies, the use of stems appeared to be beneficial for biomechanical implant fixation. Conclusions of whether more or less bone flexibility is beneficial in an AORI Type 1 defect cannot be definitively drawn from the flexibility results of this study.

### Discussion (2) If short stems are used instead of long stems, proximal implant fixation will increase and load behavior will most closely resemble the behavior of an intact bone

In this study, increased stem length reduced distal relative micromotions independent of implant loading, offering a better initial state for osseointegration in the distal areas; however, these results were accompanied by increased proximal relative motions in the case of the varus-valgus load. Loads of up to three times greater than during axial rotation can occur during varus-valgus torque in knee implants [35], increasing the significance of these results. With increased stem length, the main fixation areas were slightly moved to the diaphysis, which could also increase the risk of metaphyseal stress shielding.

Regarding bone flexibility, increased stem length appears to stiffen the bone distal to the end of the shorter stem. Using a stem, also appears to stiffen the bone along the implant and increase bone flexibility of the bone distal to the implant compared to an intact bone.

In comparison to the current study, Conlisk et al. [42] observed varying effects on implant fixation and component stability depending on stem length. They compared the stem length in a cemented and un-cemented femoral TKA in a biomechanical Sawbone^®^ model. A similar increase of distal fixation could be observed when using longer cementless stems. This correlates with the results of Completo et al. [44], which measured an increased load sharing along the stem when using long press-fit stems in addition to a tibial tray. However, no metaphyseal support with sleeves was used in this study. McLean et al. [45] investigated the effect of stem length on micromotions of tibial revision implants in a synthetic bone model. Although a more stable proximal fixation was measured for implants without stems or with short stems, a reduced subsidence of the implant was shown when using long stems.

Considering these results, using implants with sleeves and shorter stems appears to be beneficial for overall implant fixation, especially in the presence of varus-valgus loads.

### Discussion (3) If stems with undersized diameters are used instead of press-fit stems, load behavior will most closely resemble the behavior of an intact bone

In the current study, undersizing of stem diameter reduced proximal relative micromotions and simultaneously increased relative motions in the distal area of the tibial tray. The findings confirm that the undersized stems will support the proximal fixation of metaphyseal sleeves. Both stem options appear to stiffen the bone proximally and increase bone flexibility distal to the implant. In addition, the press-fit stem group matches the bending of an intact bone more closely around the distal end of the implant. The less stiff, undersized stem increases implant-bone-flexibility. This increased flexibility does not resemble the bending of the intact within this study. However, reduction of stem stiffness (e.g. smaller diameter, other material properties, use of distally slotted stems) may help to resemble the flexibility of an intact bone in case of stiffer, implant-bone compounds (e.g. with long stems as shown in hypothesis 2).

Jazrawi et al. [46] analyzed the effects of press-fit stem size and length on implant stability in a paired cadaver study, showing decreased tray motion with increased stem length and increased stem diameter, which is somewhat similar to this study’s interpretation. However, Jazrawi et al. [46] did not measure relative micromotions along the length of the stem and thus could not conclude on the fixation of the complete implant. If only the relative motion at the tibial tray could have been analyzed, no difference would have been observed regarding stem diameter and length. The additional metaphyseal sleeve fixation could be a possible cause for this and shows that the effect of stem stiffness on implant stability is less dominant if a sleeve is present.

In the presence of a moderate AORI Type T1 bone defect, the use of press-fit stems compared to less stiff undersized stems appears to more closely resemble the flexible behavior of an intact bone. Variation of stem stiffness may be used to increase implant-bone-flexibility in the case of more rigid implant-bone compounds.

### Limitations

Limitations of this study include the use of synthetic bone and the surgeon himself as a potential variable. High methodical standardization reduced many influencing factors (e.g. bone defects, morphologies, implant size, patient specific preoperative conditions, implant loading, etc.). Most of these factors were eliminated with the use of standardized synthetic bones. Although biomechanical comparability of synthetic bones and human specimens has been proved by Cristofolini et al. [47], long-term implant fixation as a result of biological processes cannot be analyzed. Therefore, only initial implant fixation was measured. Unfortunately, some factors could not be standardized, such as the preparation of bones, which was performed by an orthopedic surgeon instead of robotically reaming and rasping. Although a linear correlation between axial implant load and relative motion is known [30–33], the use of varus-valgus loads, which were reduced from physiological values, is another limitation of this study.

This study focused on the biomechanical behavior of different cementless stem options in combination with tibial knee implants; however, the use of cemented short stems to treat tibial defects is quite common. Cement fixation could affect implant stability and change the flexibility of the tibia. Therefore, excluding cemented stems limited the study. The DIC system itself is also limited. The position of each measuring point has to be calculated from two different cameras which might result in differences of precision and accuracy in the frontal plane (point of interest is not in the optical axis) and the depth plane (depth of field is poorly focused). The DIC system was validated with a high resolution coordinate measuring machine and calibrated with a measuring volume, which was supposed to be larger than the region of interest during motion. Nevertheless, if intensive out of plane motions were to appear, then some measurement points may not be exactly located. It would then have to be validated that these points did not adversely affect the resulting data. Additionally, the problem of distinguishing between combined elastic motions and plastic deformations should be addressed when analyzing relative micromotions, although it typically receives more attention in long-time load tests. If the implant-bone-fixation is weak, an implant could migrate during load application, and this permanent migration would affect the measured relative motions.

## Conclusion

This study shows that using tibial revision implants with stems and large sleeves vs. only large sleeves could be beneficial for initial implant fixation. Whether a simultaneously increased risk of proximal stress shielding for implants with stems is dominant compared to the advantage of better initial fixation still needs to be investigated. In combination with metaphyseal sleeves, longer stems appear to offer a better initial state for osseointegration, but also introduce the risk of proximal stress shielding. As short and long stems offer micromotions below the threshold for osseointegration, utilizing short stems in Type I defects may offer a compromise between better osseointegration and less proximal stress shielding. Stem stiffness appears to have some relationship with bone flexibility. Stiffer, short stems appear to follow the bending of the intact bone most closely; however, further investigation is needed before concluding whether stiffer short stems or less stiff longer stems are more favorable for this application.

## Supporting information

S1 Table Rotational and bending implant stability results of all groups. (PDF)

S2 Table Implant-bone flexibility results of all groups. (PDF)
