# Supplementary material for: Tibial revision knee arthroplasty with metaphyseal sleeves: The effect of stems on implant fixation and bone flexibility
Source: PLoS One. 2017 May 8;12(5):e0177285. doi: 10.1371/journal.pone.0177285 (PMC5421801; doi:10.1371/journal.pone.0177285)
Supplement: S1 Table — (PDF) [file pone.0177285.s001.pdf]

IMPLANT STABILITY

Group		Relative micromotions in µm																	
		rotational torque									Varus-valgus torque								
		#1	#2	#3	#4	#5	#6	#7	#8	#9	#1	#2	#3	#4	#5	#6	#7	#8	#9
A	n°1	7	5	3	3	6	7	16	25		14	12	5	4	2	2	9	18	
	n°2	7	5	3	3	6	9	17	23		14	12	5	4	2	1	7	16	
	n°3	5	3	3	3	5	6	14	23		15	12	5	4	2	2	10	19	
	n°4	8	5	1	2	4	5	13	22		16	13	5	4	2	2	10	21	
	mean	7	5	2	3	5	7	15	23		15	12	5	4	2	2	9	18	
	±SD	*1*	*1*	*1*	*1*	*1*	*2*	*2*	*1*		*1*	*1*	*0*	*0*	*0*	*0*	*1*	*2*	
B	n°1	4	4	5	5	7	8	15	23		18	15	6	5	2	2	12	23	
	n°2	4	3	5	5	7	8	15	24		14	12	5	4	2	2	8	17	
	n°3	4	2	5	5	7	8	15	23		18	14	6	5	2	2	10	21	
	n°4	4	3	4	4	6	7	14	23		16	13	5	4	1	1	10	20	
	mean	4	3	5	4	7	8	15	23		17	14	5	5	2	2	10	20	
	±SD	*0*	*1*	*0*	*0*	*0*	*0*	*0*	*1*		*2*	*1*	*1*	*1*	*0*	*0*	*1*	*2*	
C	n°1	6	5	2	2	4	4	11	18	26	15	14	9	8	6	5	3	8	15
	n°2	8	6	2	2	3	4	11	19	28	14	12	8	7	5	5	2	7	13
	n°3	6	5	2	2	3	4	10	18	26	14	13	8	8	6	5	2	7	13
	n°4	6	4	1	1	3	3	10	16	24	14	12	8	7	5	5	3	8	14
	mean	7	5	2	2	3	4	10	18	26	14	13	8	8	6	5	2	7	14
	±SD	*1*	*1*	*0*	*0*	*0*	*0*	*1*	*1*	*2*	*1*	*1*	*1*	*1*	*0*	*0*	*0*	*1*	*1*
D	n°1	5	5	13	14	18					23	17	6	8	17				
	n°2	6	9	19	19	24					26	19	9	11	22				
	n°3	8	4	10	12	16					22	16	4	5	11				
	n°4	4	3	10	11	15					21	16	5	7	14				
	mean	6	5	13	14	19					23	17	6	8	16				
	±SD	*1*	*2*	*4*	*4*	*4*					*2*	*1*	*2*	*2*	*4*				
