# Supplementary material for: Tibial revision knee arthroplasty with metaphyseal sleeves: The effect of stems on implant fixation and bone flexibility
Source: PLoS One. 2017 May 8;12(5):e0177285. doi: 10.1371/journal.pone.0177285 (PMC5421801; doi:10.1371/journal.pone.0177285)
Supplement: S2 Table — (PDF) [file pone.0177285.s002.pdf]

IMPLANT-BONE FLEXIBILITY

Group		Varus-valgus torque: Relative micromotions in µm									
		Lateral load					Medial load				
		$\Delta_{b1,b2}$	$\Delta_{b2,b3}$	$\Delta_{b3,b4}$	$\Delta_{b4,b5}$	$\Delta_{b5,b6}$	$\Delta_{b1,b2}$	$\Delta_{b2,b3}$	$\Delta_{b3,b4}$	$\Delta_{b4,b5}$	$\Delta_{b5,b6}$
A	n°1	-2	-3	-9	-7	-8	-1	2	6	6	9
	n°2	-3	-4	-8	-6	-10	1	4	8	7	8
	n°3	-2	-6	-12	-9	-11	3	7	14	10	12
	n°4	-6	-7	-14	-12	-13	3	6	13	11	14
	mean	-3	-5	-11	-8	-10	1	5	10	9*	11*
	±SD	*2*	*2*	*3*	*3*	*2*	*2*	*2*	*4*	*2*	*3*
B	n°1	-3	-9	-16	-12	-13	2	8	15	12	13
	n°2	-2	-6	-11	-8	-11	2	7	8	9	12
	n°3	-6	-8	-15	-11	-13	3	8	15	10	16
	n°4	-6	-5	-10	-6	-8	4	5	9	7	8
	mean	-4	-7	-13*	-10	-11	3	7	12*	9*	12*
	±SD	*2*	*2*	*3*	*3*	*3*	*1*	*1*	*4*	*2*	*3*
C	n°1	-2	-2	-2	-4	-7	1	2	5	2	4
	n°2	0	-1	-2	-5	-5	0	0	4	2	5
	n°3	-4	-5	-5	-7	-8	2	3	5	4	5
	n°4	-2	-6	-6	-8	-9	1	3	6	6	7
	mean	-2	-3*	-4*	-6	-7	1*	2*	5	3	5
	±SD	*2*	*2*	*2*	*2*	*1*	*1*	*1*	*1*	*2*	*1*
D	n°1	-7	-4	-6	-2	-5	4	4	3	2	2
	n°2	-7	-7	-4	-4	-4	5	2	6	3	6
	n°3	-6	-5	-5	-4	-6	3	5	4	3	6
	n°4	-6	-6	-7	-6	-6	5	6	6	5	7
	mean	-6	-6	-6*	-4*	-5	4	4	5	3	5
	±SD	*1*	*1*	*1*	*2*	*1*	*1*	*1*	*2*	*1*	*2*
E	n°1	-5	-8	-9	-10	-9	2	3	6	2	3
	n°2	-6	-7	-8	-5	-7	5	7	5	3	3
	n°3	-5	-5	-6	-7	-4	3	6	6	4	5
	n°4	-3	-9	-8	-8	-10	3	5	7	5	3
	mean	-4	-7	-8	-7	-7	3	5	6	4	4
	±SD	*1*	*2*	*1*	*2*	*2*	*1*	*2*	*1*	*1*	*1*
